# Coral Growth and Bioerosion of *Porites lutea* in Response to Large Amplitude Internal Waves

**DOI:** 10.1371/journal.pone.0073236

**Published:** 2013-12-09

**Authors:** Gertraud Maria Schmidt, Claudio Richter

**Affiliations:** Bentho-Pelagic Processes, Alfred Wegener Institute, Helmholtz Centre for Polar- and Marine Research, Bremerhaven, Germany; University of New South Wales, Australia

## Abstract

The Similan Islands (Thailand) in the Andaman Sea are exposed to large amplitude internal waves (LAIW), as evidenced by i.a. abrupt fluctuations in temperature of up to 10°C at supertidal frequencies. Although LAIW have been shown to affect coral composition and framework development in shallow waters, the role of LAIW on coral growth is so far unknown. We carried out a long-term transplant experiment with live nubbins and skeleton slabs of the dominating coral *Porites lutea* to assess the net growth and bioerosion in LAIW-exposed and LAIW-protected waters. Depth-related, seasonal and interannual differences in LAIW-intensities on the exposed western sides of the islands allowed us to separate the effect of LAIW from other possible factors (e.g. monsoon) affecting the corals. Coral growth and bioerosion were inversely related to LAIW intensity, and positively related to coral framework development. Accretion rates of calcareous fouling organisms on the slabs were negligible compared to bioerosion, reflecting the lack of a true carbonate framework on the exposed W faces of the Similan Islands. Our findings show that LAIW may play an important, yet so far overlooked, role in controlling coral growth in tropical waters.

## Introduction

The waxing and waning of coral reefs is determined by two antagonistic processes: the accretion and erosion of calcium carbonate (CaCO_3_). Scleractinian corals are the most important contributors to the reef framework [1]. Their fast-growing and large CaCO_3_ skeletons provide the building blocks for the reef, which are reinforced and kept in place by other calcifying organisms, notably calcareous algae, long after the coral colony has died [1], [2]. The largely biogenic accretion of carbonate is kept at bay by a number of physical, chemical and biological processes contributing to the dissolution and erosion of the carbonate rock. Bioeroding organisms, notably boring sponges, excavating fish, and invertebrate grazers, play a dominating role in the destruction of the carbonate [2]–[5]. The balance of accreting and erosive processes determines the net growth of the corals and coral reef framework [6], [7]. In warm, clear, aragonite-rich and nutrient-poor coral reef waters, carbonate accretion exceeds erosion and the reef framework grows [2]–[4], [6], [8]. Less aragonite supersaturated, turbid and nutrient-enriched waters directly or indirectly constrain carbonate accretion [9]–[11] and favour carbonate dissolution [12], [13] and bioerosion [14], [15], tipping the carbonate balance. Low pH conditions coinciding with a shift in the carbonate chemistry and reduced aragonite saturation can exceed the energetic demands of corals for the maintenance of calcification [9], [16] resulting in reduced coral growth or even dissolution [12], [13]. High nutrient concentrations favour the growth of phytoplankton and filter-feeders, many of which are internal bioeroders [2], [14], [15], [17]. They also foster the growth of benthic macroalgae which are an important food source for excavating grazers [2], [18]. Hence, nutrient-enhanced bioerosion is predominantly found in areas influenced by river runoff [8], [17] and upwelling [19], [20].

Recent research mainly focuses on anthropogenic factors threatening the health of corals and coral reefs. The effect of decreasing pH and aragonite saturation [9], [12], [13], [21], increasing temperature [22] and dissolved nutrient loads [23], [24] endanger coral reefs worldwide [25]–[27]. In a rapidly changing environment, corals will be exposed to stronger environmental changes within shorter periods of time (years to decades) with lower chances to acclimatize or adapt [25], [27]. Naturally fluctuating environments provide an opportunity to study corals under conditions similar to future projections of global warming and/or ocean acidification [3], [4], [11]–[14] offering insight into metabolic tolerances and acclimation potentials of corals to extreme conditions. There is mounting evidence that corals exposed to fluctuations in water chemistry (i.e. pH, nutrient concentrations) and/or temperature are more resistant to heat stress ([28], [29] and references therein), and it is possible that corals subjected to natural variations in their physico-chemical environmental may show pre-adaptation potential to cope with climate change.

In this regard, a particular phenomenon occurring in many tropical areas are non-linear large amplitude internal waves (LAIW) occurring at supertidal frequencies (several waves per tidal cycle) [30]–[32]. LAIW may entrain sub-pycnocline waters into shallow reef areas [31], [32] and cause dramatic changes in environmental conditions which exceed by far the daily and seasonal fluctuations in temperature, aragonite saturation and nutrient concentrations in coral reefs [33]. While low magnitude variations at tidal frequencies have shown mixed effects on coral calcification [34], the influence of LAIW on coral growth, calcification and bioerosion has not been studied yet, in spite of their widespread occurrence [35].

The Similan Islands, an archipelago off the west coast of Thailand in the Andaman Sea, are exposed to LAIW. The sub-pycnocline deep water causes sudden (within 1–2 minutes) drops of up to 10°C in temperature, of 0.6 units in pH and increases of up to 9.4 µmol in NO_x_ l^−1^ [31]. LAIW impact on these reefs varies seasonally. LAIW are stronger during the calm northeast (NE) monsoon (January through April) [31]. During the southwest (SW) monsoon (May through October) strong winds cause mixing and resuspension of bottom sediments [32]. Both LAIW and SW monsoon act from the same W to SW direction. Hence, in the Similan Islands, ocean facing W reefs are exposed to both monsoon and LAIW, while the east (E) reefs are sheltered from both phenomena. As a result, the islands feature a peculiar reef development which is restricted to the E sides, whereas a true framework is lacking on the W sides [31], [32], [36]. The development is in contrast to most other corals reefs, where growth is most pronounced on the windward sides [37], except in upwelling areas where coral growth appears to be suppressed in a similar fashion [28]. Although the observed asymmetry has been attributed to the combined effects of monsoon and LAIW [31], [32], experimental evidence for a reduction in coral growth is so far lacking. Parallel studies showed a higher biomass, protein content and nutritional status in exposed corals [38], [39], and a better survival during periods of restricted photosynthesis [38]. This could suggest a potentially higher energy allocation to calcification. On the other hand, calcification may be more energy-demanding and therefore reduced [9], [40], due to the frequent exposure to low pH waters [31]. The combination of seasonally enriched nutrient conditions and enhanced exposure to storm and high wave energy along the W areas might further undermine the development of a stable carbonate reef framework as coral growth may not compensate for both, framework destruction by bioerosion and wave energy [41].

The present study explores coral growth and bioerosion on dead skeletal substrate under marked differences in LAIW and monsoon exposure. We hypothesize that LAIW and monsoon differentially affect shallow (∼7 m), deep (∼20 m), west (W) and east (E) carbonate production and erosion, representing areas of strong monsoon (W 7 m), strong LAIW (W 20 m), moderate (E 20 m) and low (E 7 m) LAIW impact, respectively. Coral growth may be reduced at the exposed W compared to the sheltered E due to the combined effect of low temperature, low pH and high nutrients depressing growth [42]–[45]. We hypothesize further that bioerosion will be enhanced due to nutrient stimulated boring filter-feeders [18], [46] and increased grazing by excavating herbivores taking advantage of nutrient-enhanced macroalgal growth [23]. Other calcifiers (collectively referred to “fouling community” in the following) may on the one hand benefit from LAIW-enhanced supply of food [38], but also suffer from reduced CaCO_3_ precipitation rates.

## Materials and Methods

### Experimental setup

The massive coral *Porites lutea* (Milne Edwards and Haime 1851) was used as a model organism for this study. It is the dominant reef-building coral in the Thai Andaman Sea [36], [47], [48] and the coral of choice for numerous physiological and ecological investigations in the Andaman Sea and elsewhere [47]–[49]. In addition *P. lutea* in this region was shown to be associated with the same algal symbiont (C15) [50] excluding possible symbiont-related differences in growth. Coral growth in this study is defined as the net calcium carbonate accretion of a living, healthy and unscathed coral nubbin. Although neither biological erosion due to excavating grazers or boring filter feeders nor biological dissolution due to microbial activities or endolithic algae can be ruled out, we consider these factors negligible in a healthy coral [2], [51]. Bioerosion is the carbonate loss due to biological processes. It can be determined from the post-exposure net mass loss of carbonate blocks by subtracting mass accretion due to fouling organisms. Carbonate dissolution in aragonite saturated waters with pH>7.4 can be ruled out [13]. The pH drops due to LAIW are only intermittent (15 to 30 minutes) and usually above the critical value [31].

**Figure 1:**
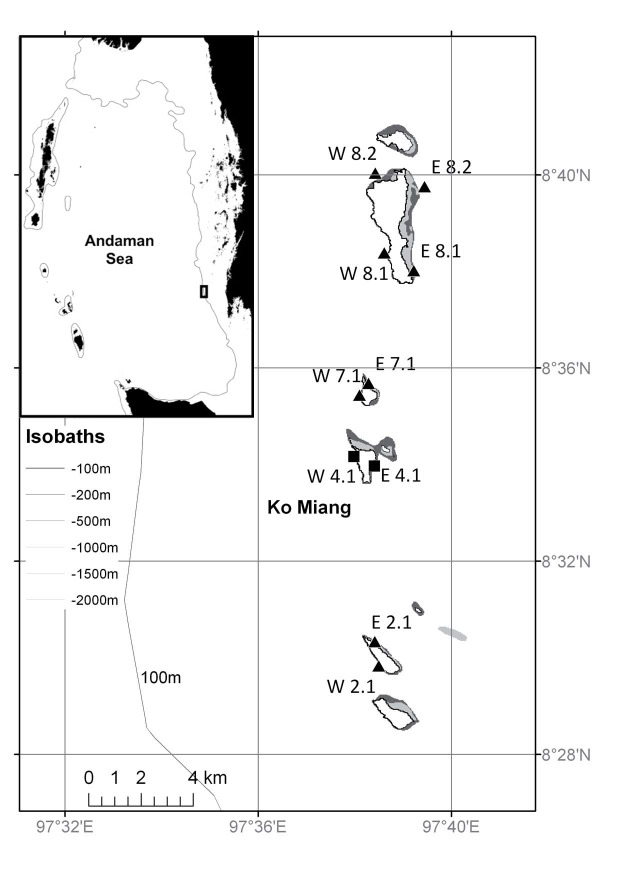
Locations of study sites in the Similan Islands, Andaman Sea, Thailand. Symbols show locations of temperature loggers and growth rate experiments (▴) and of bioerosion experiments (▪). (Figure inset courtesy A. Buschmann, AWI. Data source: http://www.ngdc.noaa.gov/mgg/shorelines/gshhs.html).

To investigate the effect of LAIW and monsoon on coral growth and bioerosion, coral nubbins and coral carbonate blocks from a shallow (7 m) sheltered (E) site were transplanted to 20 Similan Island sites: shallow (7 m, monsoon effect) and deep (20 m, LAIW effect) sites on the exposed (W) and sheltered (E) sides of 5 different locations in the Similan Islands archipelago (Fig. 1). Despite the limitations of such a one-level experimental design with all experimental coral nubbins originating from the same location in contrast to a complete cross-transplantation design, sampling restrictions in the National Park did not allow for another approach. But the results indicate (see below) that coral origin had no significant effect on mortality (Table S1, S2), as opposed to findings in other shallow to deep transplant experiments [52]. We assessed transplanted (E 20 m, W 7 m, W 20 m) and control growth (E 7 m) and bioerosion after 12 and 21 months exposure periods between February 2007 and November 2008, coinciding with high and low LAIW activity years [39]. The study in the Similan National Park was carried out with the official research permit of the National Research Council of Thailand (NRCT, permit numbers: TS0907.1/12593 and NRCT002.3/03231).

### Temperature

Temperature loggers (TidbiT v2, Onset computers; resolution 0.2°C) collecting data at 6 min intervals were deployed along with the transplants. Intercalibration of the TidbiT loggers with a high precision digital thermometer (Amarell ama-digit ad 3000 th) showed low deviations between instruments (<0.3°C) compared to the up to 10°C variations measured within the respective instrument records (see results). Because the arithmetic mean is sensitive to extreme values, temperature data are reported as the most frequent (or mode) values along with the corresponding ranges. As a measure for LAIW frequency and intensity, we integrated the temperature anomalies (measured value versus daily mode temperature) over time yielding a cumulative cooling index in degree days (DD in [°C d], cf. [34]). The temperature anomalies were multiplied by the sampling interval in days (the sampling interval of 6 minutes equaled to a 0.0042 interval in days). A value encapsulating LAIW impact was obtained by summing up all negative temperature anomalies for each time period of interest (12 months: February 2007 to February 2008 and 21 months February 2007 to November 2008; see below).

### Coral growth rates

For the growth experiment, a total of 200 *Porites lutea* nubbins was collected chiseling 20 nubbins from each of 10 large mother colonies at the sheltered E side of the Similan island Ko Similan (8°38′21.43″N 97°38′59.73″E) from 7 m depth. The nubbins were transferred to a laboratory where they were kept in flow-through reef water aquaria for 1 day to measure and weigh them. Growth rates were determined gravimetrically (cf. buoyant weight technique [53]) using a high precision microbalance (Sartorius ME-235S, precision 0.01 mg, Fig. S1) by comparing the weights of each individual nubbin before and after the 12 month growth period. Initially, *P. lutea* nubbins were measured twice, before and after attaching them to individual numbered holders using fast drying concrete (Fig. S2). The weight of each nubbin was recorded along with the corresponding weight of the nubbin attached to its holder. We used a skeletal density of 1.41 g cm^−3^ for *P. lutea* determined after Davies [53] to calculate the dry mass of each nubbin. Coral nubbins were approximately 3 to 5 cm in diameter, and had a mean mass of 42.9 g (Table S2).

**Figure 2:**
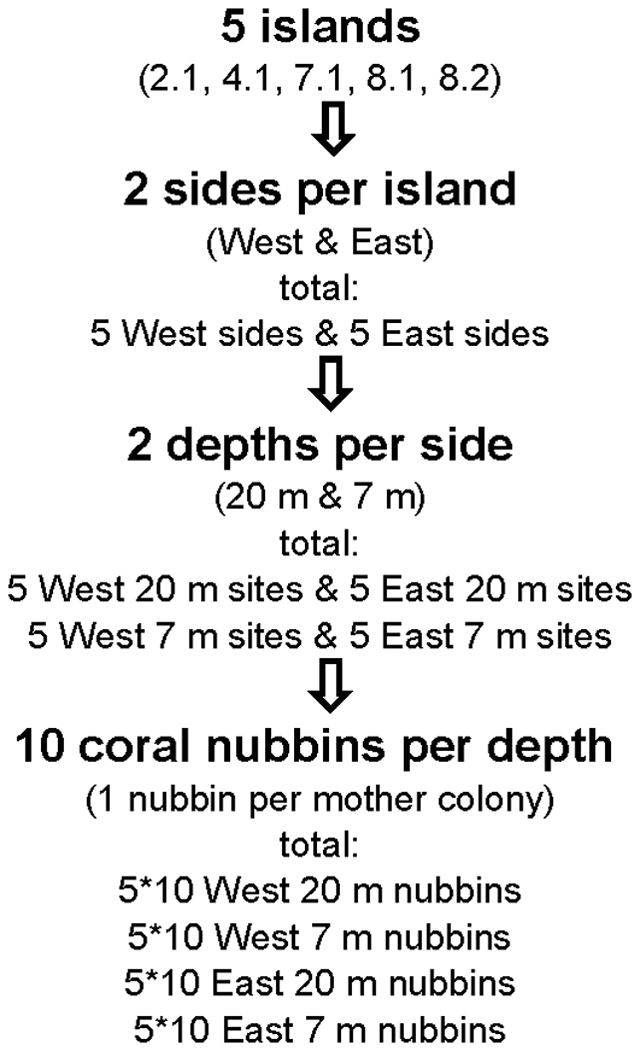
Experimental design of *Porites lutea* growth rate experiment. For the experiment in each case 20 coral nubbins were collected from 10 mother colonies at a sheltered east site in 7(close to E 8.1, cf. Fig. 1) and transplanted to the west and east sides of 5 islands at 2 depths (7 m and 20 m) from February 2007 to February 2008 along the Similan Islands (cf. Fig. 1).

The nubbins were distributed in a way that each of the 20 sites (5 locations×2 sides×2 depths, Fig. 1) received 1 representative of all 10 mother colonies. Nubbin sizes were randomized to avoid bias (Fig. 2). Hence the resulting 10 nubbins per site represented one genetically identical population. By this a systematic error due to possibly existing cryptic species [54] within the original *P. lutea* community could be avoided. The nubbins, each one on its individual holder, were attached to acrylic racks (Fig. S2) fixed on dead coral substrate. The experiment was started between February 20th and 24th 2007 and ended between February 22nd and 27th 2008. Logistic constraints did not allow revisiting the sites on a regular basis for maintenance. To avoid artefacts due to fouling, the nubbins were therefore left unprotected, even though this increased the risk of losing nubbins due to fish predation [55] or waves. The high number of nubbins deployed ensured, however, a sufficient number of replicates for statistical analyses after the exposure period (Table S2). At the end of the exposure period the corals were retrieved and returned to the laboratory. Dead nubbins covered with fish bite marks where discarded. Only undamaged and living nubbins were used for the analysis. Nubbin holders were cleaned of epiphytes and the nubbins on their holders weighed, first in seawater and subsequently in air after drying at 60°C for 24 h. Growth rates (g yr^−1^) were calculated as change in mass over the 1 year duration of the experiment, assuming no significant mass change in the plastic holders which were free of bite marks after the exposure. There were no differences in the initial nubbin weights between exposure treatments (side and depth, Student's t-tests, Table S3). Assuming no significant size-dependent differences in area-specific growth [56] we normalized the final weights to the initial weights and expressed the mean growth rates as % yr^−1^.

### Bioerosion rates

Bioerosion was assessed by deploying bioerosion-free dead coral skeleton and measuring the weight differences before and after the exposure. A total of 72 rectangular blocks of 8×8×1.5 cm^3^ were cut from the skeleton of a dead *P. lutea* colony (Fig. S3) collected from the fringing reef close to Phuket Marine Biological Centre at the southern tip of Phuket peninsula (7°47′58.76″N, 98°24′31.14″E, Fig. 1). Only immaculately clean blocks from the inner portions of the colony were selected. The blocks were soaked in running fresh water, dried, perforated in the center with a 5 mm drill for later attachment, measured to the closest mm, and weighed to the closest 0.01 g. Groups of blocks were mounted on PVC racks yielding a total of 12 racks holding 6 blocks each. For each rack, blocks were fixed at a distance of 15 cm to each other and 2 cm to the rack (Fig. S4), using stainless steel screws and bolts. Limitations on the amount of material restricted the spatial scale of the bioerosion experiment to the eastern and western side of the central Similan island Ko Miang (Fig. 1). Previous studies showed the island to represent very well the general oceanographic and reef ecological conditions of the Similan Island archipelago, reflecting the environmental conditions, coral cover and framework distribution [31]. At each of the 4 sites (W×E, 20×7 m), 3 racks were attached to dead coral substrate in a distance of 20 m to each other. The racks were deployed mid of March 2007. Half of the blocks (i.e. 3 blocks per rack) were retrieved after 12 months (mid of March 2008), the remainder after 21 months (mid of November 2008). The block numbers to be sampled were chosen randomly before collection to avoid personal bias. Immediately after collection the blocks were bleached to remove organic material, rinsed in fresh water, dried and weighed as above. Care was taken not to lose any material during handling. Carbonate accretion due to calcareous fouling organisms (serpulids, bivalves, balanids and corals growing particularly on the undersides of the blocks, cf. Fig. S5, S6) was calculated from their skeletal volumes (cm^3^) and respective skeletal densities taken from the literature [47], [48], [53], [57] (Fig. S5). Volumes were estimated from length measurements carried out under a stereomicroscope applied to geometric approximations of the skeletal structures. Tubes of serpulids and balanids were represented by truncated hollow cones with the tube walls accounting for 40% of the tube volume, respectively [57]. The volume of bivalve shells was calculated by multiplying the area of the shell (approximated by the planar projection of the shell on a surface) with the shell thickness. The volume of small dome-shaped corals was calculated as 3/8 π r^3^, where r is the radius of the coral skeleton. For branching corals, the volume was calculated by the sum of branch segments approximated by cylinders (π r^2^ l, where l is the length of the branch segment). Serpulids, balanids and bivalves were assumed to have a skeletal density of 2.7 g cm^−3^ [39], corals' density was taken as 1.4 g cm^−3^ after Davies [53] and in accordance with previous bulk density determinations [47], [48]. Bioerosion values for both exposure periods (12 and 21 months) were normalized to one year and expressed as kg m^−2^ [5], [7].

### Statistical analysis

For statistical analyses the software Statistica v 9 was used. Data were tested for normal distribution and homogeneity of variances with Kolmogorov-Smirnov and Levene's test, and square root transformed if necessary. The effect of exposure (W and E) and depth (7 and 20 m) on rates of coral growth and bioerosion was analyzed using analysis of covariance (ANCOVA), with side and depth as the treatment factors and the initial weight of coral nubbins and dead skeleton blocks as the respective covariate. Posthoc, pair wise comparisons of the adjusted group means were performed via Tukey HSD-tests. Possible site or rack effects between coral fragments exposed at the same site on the same rack were considered by taking into account the factor site as random factor into the analysis. This factor was statistically insignificant (see results) and allowed to pool the data. The effect of exposure (W and E) and depth (7 and 20 m) on the temperature conditions (monthly mode, maxima, minima and negative cumulative temperature anomalies (calculated as degree days, °C d) was tested with a non-parametric Kruskal-Wallis ANOVA and median test followed by multiple comparisons of mean ranks. Both growth rate data and bioerosion data were related to the temperature conditions (cumulative °C d) at each site. General linear models were fitted to the data with growth rate and bioerosion as dependent and temperature as independent variables. If not stated otherwise data are displayed as means (± SE).

## Results

### Temperature

**Figure 3:**
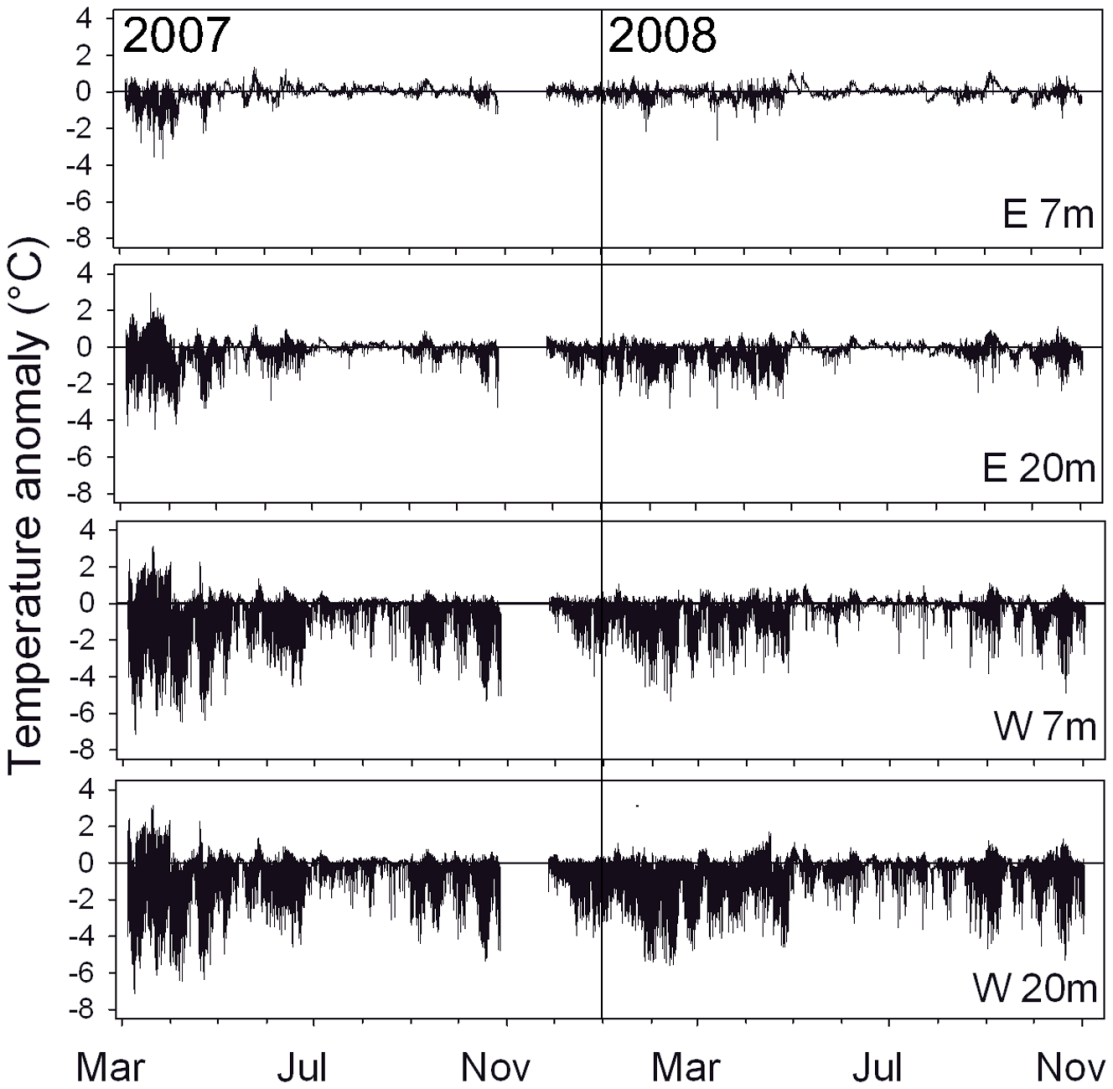
Temperature anomalies at Similan island Ko Miang. Study sites E 4.1 and W 4.1, period March 2007 to November 2008. Anomalies were calculated relative to mode values (gap in data due to exceeded storage capacity).

**Figure 4:**
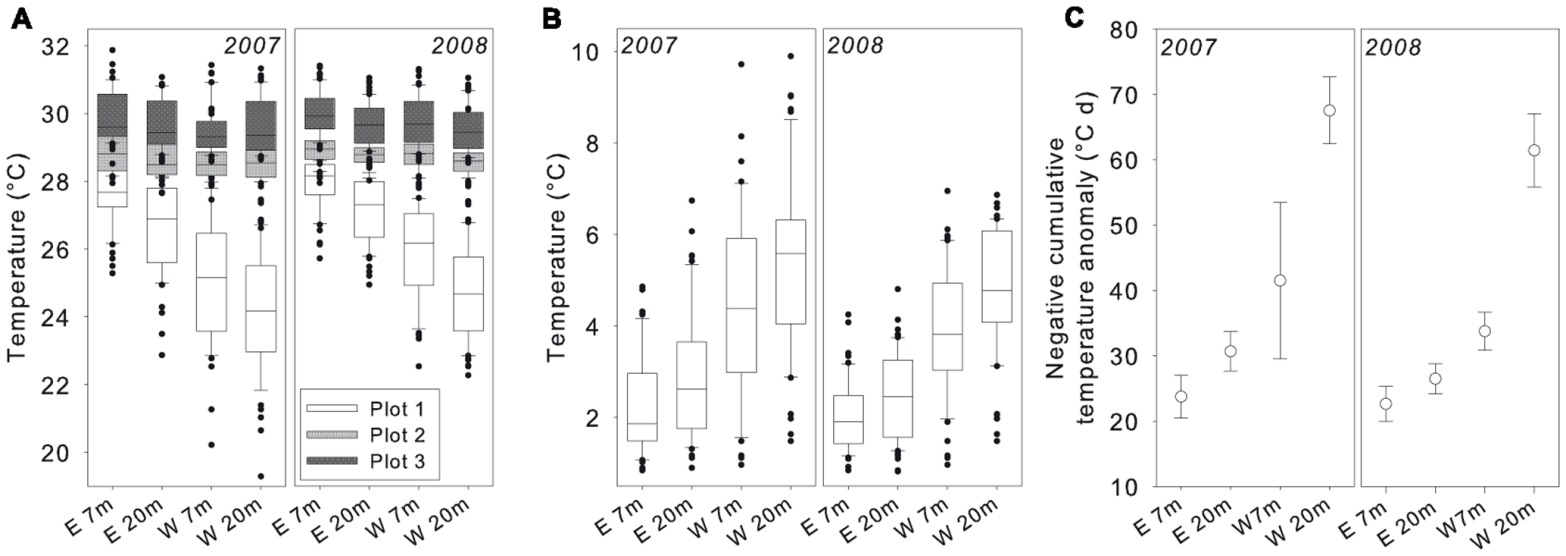
Temperature data for all Similan Islands sites. Central tendency box plots (median with 25^th^ and 75^th^ percentile and non-outlier range) with extremes (dots) (A, B) and scatter plots with standard deviation (SD, whiskers) for the study periods [Feb 2007 to Feb 2008 (*2007*) and Nov 2007 to Nov 2008 (*2008*)], sides (east, E and west, W) and depths (7 and 20 m), featuring overall monthly minimum, mode and maximum temperatures (A), monthly temperature ranges (B), and negative cumulative temperature anomalies (calculated as degree days, °C d; calculation details see methods) (C).

Temperature variations showed the strongest presence of large amplitude internal waves (LAIW) between February and April (Fig. 3) corresponding to the weak north east (NE) monsoon (e.g. W 20 m: monthly ΔT = 8.6±0.2°C in March 2007). The lowest LAIW activity was found between July and November (e.g. W 20 m: monthly ΔT = 3.8±0.3 in 2007) corresponding to the strong south west (SW) monsoon. Significant interannual differences were also evident (W 20 m: monthly ΔT = 6.0±0.1°C in 2008, i.e. a 2.6°C smaller range than in 2007) (Table 1). Temperatures varied significantly between, but not within the levels of the two factors depth (levels: 7 m, 20 m) and exposure (levels: W, E) ([31], this study]. The temperature anomalies split by years (2007 and 2008), exposure (W versus E) and depth (7 and 20 m) are shown in Fig. 4 A and B. Differences between monthly mode and maximum values were small (Fig. 4 A, mode: ΔT<3.6°C, maxima: ΔT<3.0°C) compared to the differences between mode and minimum values (Fig. 4 A, ΔT>8.0°C). Exposure and depth differences were significant for the minima (but not for modes and maxima) (Table 1) with lowest values at W 20 m (24.5±0.2°C compared to 25.6±0.2°C for W 7 m, 27.1±0.1°C for E 20 m, and 27.9±0.1°C for E 7 m) resulting in increased temperature ranges with depth and exposure, particularly in 2007 (Table 1, Fig. 3 and 4). The cumulative negative temperature anomalies which consider both, the intensity and frequency of temperature drops below the daily running mode, were most pronounced in W 20 m and decreased with decreasing exposure down to the lowest values at E 7 m (Table 1, Fig. 4 C). They did not show a statistically detectable difference between the years as found for the temperature minima and ranges (2007 and 2008).

**Table 1:** Differences in monthly temperature values at Similan Islands between depths and sides.

	df	N	Mode				maxima				Minima				range				df	N	cum. negative anomaly (°C d)			
***2007* vs *2008***	1	440	Kruskal Wallis:			n.s.				n.s.				*				*	1	40				n.s.
**2007**	3	220	Kruskal Wallis:			n.s.	Kruskal Wallis:			*	Kruskal Wallis:			***	Kruskal Wallis:			***	3	20	Kruskal Wallis:			***
				W 20 m	W 7 m	E 20 m		W 20 m	W 7 m	E 20 m		W 20 m	W 7 m	E 20 m		W 20 m	W 7 m	E 20 m				W 20 m	W 7 m	E 20 m
			W 20 m				W 20 m				W 20 m				W 20 m						W 20 m			
			W 7 m	n.s.			W 7 m	n.s.			W 7 m	n.s.			W 7 m	n.s.					W 7 m	n.s.		
			E 20 m	n.s.	n.s.		E 20 m	n.s.	n.s.		E 20 m	***	***		E 20 m	***	***				E 20 m	n.s.	n.s.	
			E 7 m	n.s.	n.s.	n.s.	E 7 m	*	n.s.	*	E 7 m	***	***	**	E 7 m	***	***	n.s.			E 7 m	***	n.s.	n.s.
**2008**	3	220	Kruskal Wallis:			***	Kruskal Wallis:			***	Kruskal Wallis:			**	Kruskal Wallis:			**	3	20	Kruskal Wallis:			***
				W 20 m	W 7 m	E 20 m		W 20 m	W 7 m	E 20 m		W 20 m	W 7 m	E 20 m		W 20 m	W 7 m	E 20 m				W 20 m	W 7 m	E 20 m
			W 20 m				W 20 m				W 20 m				W 20 m						W 20 m			
			W 7 m	*			W 7 m	n.s.			W 7 m	n.s.			W 7 m	n.s.					W 7 m	n.s.		
			E 20 m	n.s.	n.s.		E 20 m	n.s.	n.s.		E 20 m	***	***		E 20 m	***	***				E 20 m	**	n.s.	
			E 7 m	***	n.s.	n.s.	E 7 m	**	n.s.	n.s.	E 7 m	***	***	**	E 7 m	***	***	n.s.			E 7 m	***	n.s.	n.s.

Comparisons of monthly mode, maxima, minima, range = maximum – minimum values, and negative cumulative temperature anomalies (calculated as degree days, °C d). Kruskal-Wallis ANOVA and median test followed by multiple comparisons of mean ranks for all groups (year: February 2007–February 2008 (*2007*) versus November 2007–November 2008 (*2008*), side: west (W) versus east (E), and depth: 20 m versus 7 m). (df = degrees of freedom; N = sample size; p = probability level; significance levels are *0.05>P≥0.01, **0.01>P≥0.001, ***P<0.001, n.s.: not significant).

### Coral growth rate

**Figure 5:**
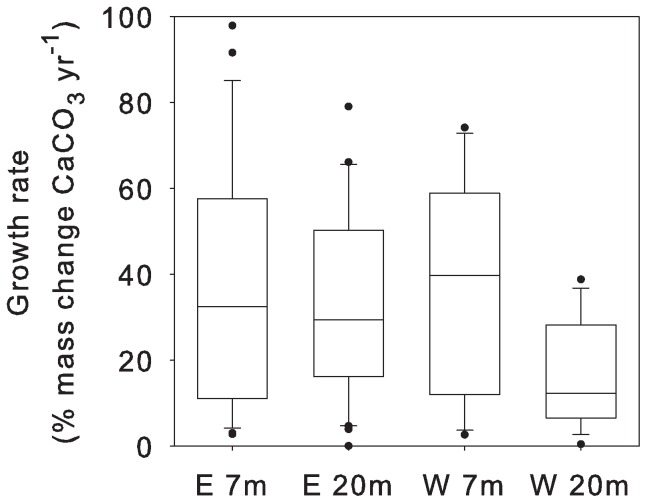
Coral growth rates (mass change of calcium carbonate, CaCO_3_) of *Porites lutea* nubbins. Nubbins were transplanted from sheltered east (E) 7 m to 7 and 20 m depth at E and west (W) sides of Similan Islands (Central tendency box plots: median with 25^th^ and 75^th^ percentile and non-outlier range, extremes: dots) between February 2007 and February 2008 (number of replicates: E 7 m: 27, E 20 m: 33, W 7 m: 13, W 20 m: 19).

ANCOVA results for the growth rates of *P. lutea* nubbins revealed significant effects of exposure and depth (p<0.038, Table 2, Fig. 5). Pair wise comparisons showed suppressed coral growth at the LAIW-impacted sites (W 20 m: 16.3±0.2% yr^−1^) compared to the monsoon-exposed (W 7 m: 36.2±0.4%) and sheltered sites (E 7 m and E 20 m: 36.7±0.2% and 36.5±0.2%, respectively, Tukey HSD, Table 2).

**Table 2:** Analysis of covariance (ANCOVA) for coral growth experiment.

Factor	df	MS	F	p
initial weight	1	3.296	3.829	*
side & depth	3	4.172	1.192	***
Site	4	0.684	0.197	0.932
side & depth*island	7	3.614	4.120	0.379
Error	76	0.861		
**Tukey HSD**, significantly different, pairwise comparisons:				p
W 20 m<E 20 m				**
W 20 m<E 7 m				**

Coral growth data square-root-transformed and analysed for east (E) and west (W) of Similan Islands from February 2007 to February 2008. Side (E and W) and depth (7 and 20 m) as treatment factors, and initial weight of coral nubbins as covariate; factor site (rack) included as random factor; posthoc, pair wise comparisons of the adjusted group means via Tukey HSD-tests. (df = degrees of freedom; MS = means square; F = F-value; p = probability level, significance levels are *0.05>P≥0.01, **0.01>P≥0.001, ***P<0.001).

### Rates of bioerosion

**Figure 6:**
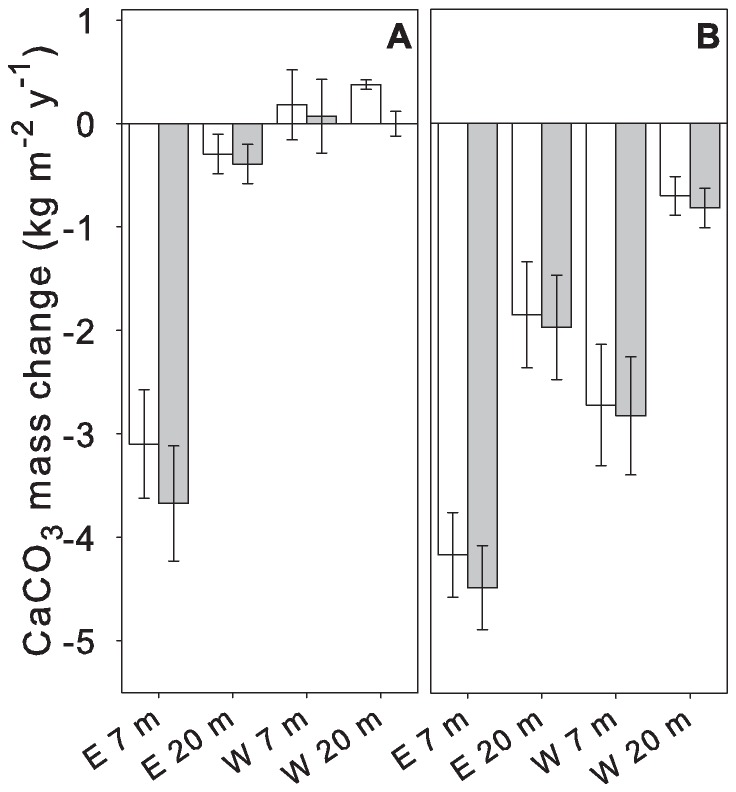
Calcium carbonate (CaCO_3_) mass change accretion (>0) and bioerosion (<0). Measured mass change (white columns) and calculated mass change (grey columns, corrected for accretion due to fouling organisms) of CaCO_3_ blocks at east (E) and west (W) side of Similan island Ko Miang. Error bars: ±1 SE of mean. Results from 12 months exposure (February 2007 to February 2008) (A), and 21 months exposure (February 2007 to November 2008) (B) normalized to 1 year (number of replicates in each case: 9).

ANCOVA results for bioerosion rates revealed significant effects of side and depth for both exposure periods (Table 3). Irrespective of exposure length, bioerosion was highest in E 7 m (Fig. 6). After 12 months exposure bioerosion was 9-fold higher in E 7 m than in E 20 m (−3.7±0.6 kg CaCO_3_ m^−2^ in 7 m compared to −0.4±0.2 kg CaCO_3_ m^−2^ in 20 m), and almost 40-fold higher than in W 7 m and 20 m where a carbonate loss was barely detectable (0.1±0.4 kg CaCO_3_ m^−2^ in W 7 m and −0.01±0.1 kg CaCO_3_ m^−2^ in W 20 m). The longer exposure (21 months) caused no change in the annual bioerosion in E 7 m (−4.2±0.4 kg CaCO_3_ m^−2^), but resulted in a more than 4-fold increase in E 20 m (−1.9±0.5 kg CaCO_3_ m^−2^), and a pronounced carbonate loss in W (W 7 m: −2.7±0.6 kg CaCO_3_ m^−2^ and W 20 m: −0.7±0.2 kg CaCO_3_ m^−2^, respectively). Again, erosion rates were highest in E 7 m (2-fold higher than in E 20 m and 5-fold than in W 20 m, respectively), followed by W 7 m with 3-fold higher erosion compared to W 20 m (Table 3 B). Biological carbonate accretion on the skeletal blocks was negligible compared to the net carbonate loss irrespective of exposure time, except for W 20 m after 12 months (net carbonate loss versus gross mass change: Student's t-test: p<0.01, Table S4, Fig. 6, Table S5, S6). Compared to the negligible 12 months exposure erosion rates in W carbonate accretion was comparatively high (Fig. 6). In all other cases the differences between net carbonate loss (corrected for the accretion due to fouling organisms) and gross mass change was less than 25% (24.3% in E 20 m after 12 months), in most cases between 4 and 7% (at E 7 and 20 m and W 7 m after 21 months exposure, Fig. 6, Table S4, S5, S6).

**Table 3:** Analysis of covariance (ANCOVA) for bioerosion experiment.

A	12 months exposure				
	Factor	df	MS	F	p
	initial weight	1	19.498	91.636	***
	Side	1	5.901	27.732	***
	depth	1	2.933	13.785	***
	side*depth	1	3.794	17.830	***
	Error	31	0.213		
	**Tukey HSD**, significantly different, pairwise comparisons:				
	E 7 m<E 20 m				***
	E 7 m<W 7 m				***
	E 7 m<W 20 m				***

B	21 months exposure				
	Factor	df	MS	F	p
	initial weight	1	36.112	33.604	***
	Side	1	12.215	11.367	**
	depth	1	20.267	18.859	***
	side*depth	1	0.526	0.489	0.489
	Error	31	1.075		
	**Tukey HSD**, significantly different, pairwise comparisons:				
	E 7 m<E 20 m				***
	E 7 m<W 20 m				***
	W 7 m<W 20 m				**

Bioerosion data (corrected for bioaccretion) square-root-transformed and analysed for east (E) and west (W) of Similan island Ko Miang. Side (E and W) and depth (7 and 20 m) as treatment factors, and initial weight of skeletal substrates as covariate; posthoc, pair wise comparisons of the adjusted group means via Tukey HSD-tests. (df = degrees of freedom; MS = means square; F = F-value; p = probability level, significance levels are **0.01>P≥0.001, ***P<0.001). Results for experimental period from February 2007 to February 2008, 12 months (A), and from February 2007 to November 2008, 21 months (B).

### Coral growth and bioerosion in relation to temperature anomalies

**Figure 7:**
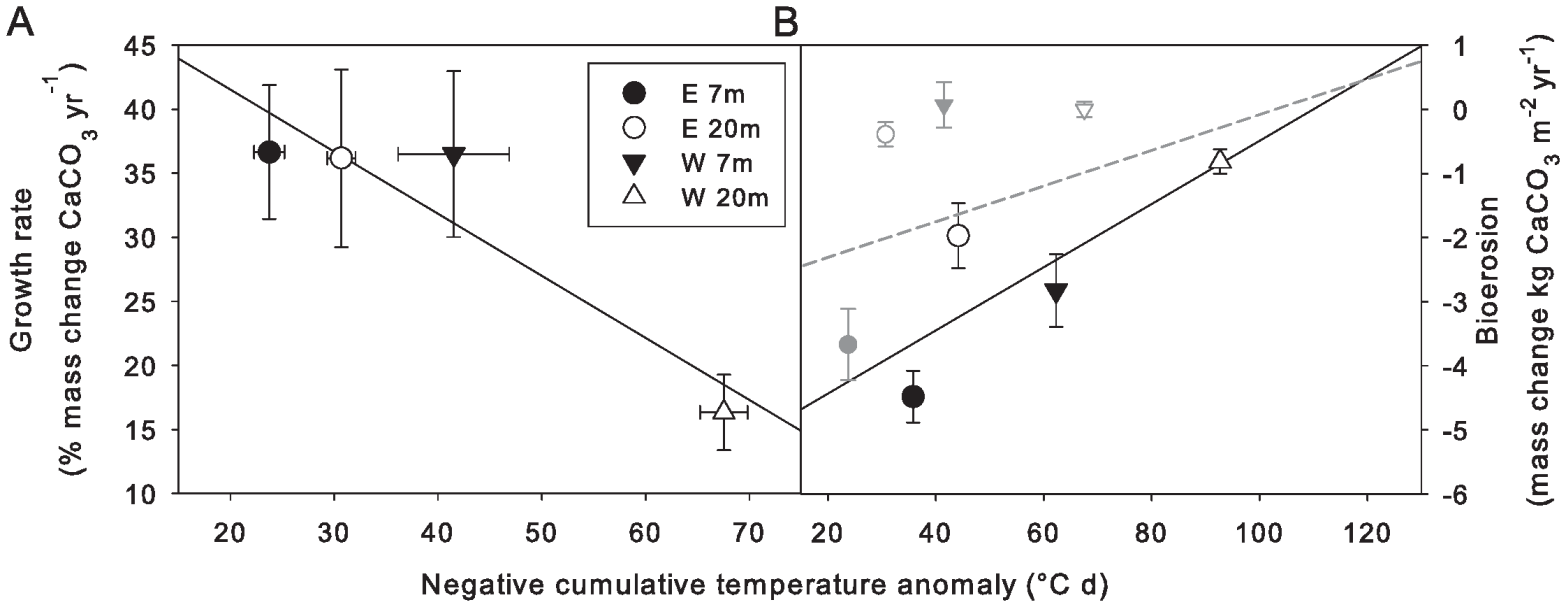
Coral growth and bioerosion as functions of temperature anomaly in west (W) and east (E), 7 m and 20 m depth. Coral growth rate of *Porites lutea* nubbins at all study sites (n = 20, df = 1, F-value = 4.4, r^2^ = 0.46, p = 0.039) along the Similan island chain after 12 months exposure (February 2007 to February 2008; see Fig. 4) (A), and bioerosion (calculated mass change in calcium carbonate (CaCO_3_) corrected for accretion due to fouling organisms) on CaCO_3_ blocks at the central Similan island Ko Miang (see Fig. 1) after 12 months (grey depiction; February 2007 to February 2008; n = 4, degrees of freedom = 1, F-value = 14.4, r^2^ = 0.30, p<0.001) and 21 months exposure normalized to 1 year (February 2007 to November 2008; n = 4, degrees of freedom = 1, F-value = 17.9, r^2^ = 0.58, p<0.001) (see Fig. 5; <0, bioerosion) (B) as functions (GLM) of the cumulative negative temperature anomaly (calculated as °C d) of the respective time period. All values are plotted as mean ± SE.

Both coral growth and bioerosion rates revealed differences between sides and depths. In order to explore the potential role of LAIW in explaining the differences, general linear models (GLM) were calculated with coral growth or bioerosion as dependent variable and temperature anomalies calculated for the respective exposure periods as independent variables. The results show that both coral growth (r^2^ = 0.46, p = 0.039) and bioerosion after 12 (r^2^ = 0.30, p<0.001) and 21 months exposure (r^2^ = 0.59, p<0.001) are inversely related to LAIW (Fig. 7).

## Discussion

### Environmental conditions

The abrupt temperature variations observed in the Similan Islands are characteristic for internal waves in coastal waters, in contrast to the much more gradual changes (hours) induced by changing tides [58]. The temperature excursions on this short time scale rival in magnitude the seasonal or monthly temperature variations at higher latitude reefs [41], [42], [59], underscoring the severity of these events and the potentially important role of cold shocks (up to 10°C in the present study) on coral metabolism and growth. The particularly strong temperature fluctuations during spring 2007 corresponded to the positive 2007 Indian Ocean Dipole (IOD) [60] with a shallow pycnocline guiding internal waves well onto the shelf, while spring 2008 corresponds to a deeper pycnocline with less pronounced temperature fluctuations around the islands. Previous studies showed the temperature drops to be associated with corresponding drops in oxygen concentrations and pH, down to 12% saturation and 0.6 units below ambient, respectively [31]. Nutrient concentrations were found to increase to up to 12-fold for nitrate and nitrite, 5-fold for silicate and over 20-fold for phosphate [31]. Although temperature variations were also measured on the E side of the islands, they were markedly lower than on the W sides of the islands (Fig. 3, 4). The environmental contrasts between W and E are also reflected in the monsoon regime causing strong swell on W but only weak wave action on the E sides of the islands [32]. Monsoon-enhanced sedimentation, surface wave action and LAIW impact were found to collectively determine reef development in the Similan Islands [32] explaining a complex, dense reef framework along the sheltered E sides contrasting the only scattered coral communities along the exposed W sides of the islands [31]. The direct relationship between LAIW intensity and depth [31] and the inverse relationship between monsoonal surface wave impact and depth [61] show that both phenomena act from opposite directions, with LAIW shaping the deeper and monsoon the shallower reef areas.

### Coral growth

The coral nubbins of *P. lutea* showed a reduced growth in W 20 m (Fig. 5, Table 2) supporting our hypothesis of LAIW suppressed growth, compared to the monsoon affected (W 7 m) and sheltered (E 7 m, E 20 m) sites. This may be due to the combined effect of unfavourable environmental conditions, notably lower temperature, light and pH in W 20 m compared to the other sites [31]. The GLM analysis shows that coral growth rates correlated well with temperature anomaly (Fig. 7), underscoring the potential role of LAIW in suppressing coral growth. The growth rates in W 7 m are important in assessing the relative importance of monsoon and LAIW for reef growth [32] by showing that monsoon exposure did *not* limit skeletal growth. It is likely that the high water motion here enhanced metabolic rates and calcification [62] and prevented a stressful permanent sedimentation on the coral surfaces.

Coral growth rates vary in response to a number of extrinsic factors, such as temperature [56], [63], aragonite saturation state [9], [12], [16] or nutrient supply [45]. Growth rates may vary even more between species [64] and within species [47] due to intrinsic factors which are so far not well understood. Early ontogeny may play an important role in determining whether skeletogenesis is slow or rapid, and it is possible that recruitment in LAIW-exposed versus –sheltered or deep versus shallow habitats may involve selection for differential growth. To test whether such a bias has occurred in our study, compromising the validity of our results in coral growth, we analysed for side- and depth-differences in another performance indicator: coral survival. It is well known that coral transplantation from shallow to deep habitats can cause coral mortality in corals that, similar to *Porites lutea*, are unable to adapt the composition of their zooxanthellae symbionts [52]. Mortalities in our study were, however, not significantly different between deep and shallow corals (Table S1, ANOVA, p>0.05), and coral growth was indistinguishable between E 7 m (source population) and deep (Table 2), suggesting that coral origin did not compromise our findings.

Coral growth depends on light availability due to the direct linkage of calcification to the photosynthetic energy supply by the zooxanthellae [40], [65]. Despite comparable light levels in W and E 20 m depths [31] (C. Jantzen, unpublished data) growth rates in E 20 m were significantly higher than in W 20 m and comparable - if not higher - than in E 7 m – in spite of 3-fold lower light levels. Differences in light levels are therefore unlikely to explain the drastic E-W differences in coral growth at 20 m - emphasizing the importance of LAIW - notably temperature and aragonite saturation.

Coral growth is strongly dependent on temperature. Extended periods of low temperature stress (<19°C) were found to affect coral photosynthesis, tissue maintenance and growth [66]. Cold temperature can reduce the photosynthetic efficiency [67] and coral calcification [63]. Although extended periods of cold temperatures are known to have a negative effect on coral metabolism and growth [42], [67], it is not known so far if and to what extent repeated short-term temperature drops affect coral physiology and growth. But the effects on corals may be similar as described in several studies on daily temperature variations in shallow habitats affecting coral metabolism and heat stress. Although these studies deal with positive temperature anomalies it is likely that the mechanisms evoked by the thermal history [68], [69] including acclimation [70] and adaptation strategies [71] are similar to corals exposed to temperature drops. The results in this study indicate that step changes in temperature conditions of 6 to 9°C at supertidal frequencies (Fig. 3 and 4) could account in large parts to the reduced growth performance of the corals in W 20 m. Calcification in corals is further known to depend on high aragonite saturation to maintain a high saturation state at the site of calcification [9], [12], [40]. The temperature related drops in pH of 0.2 to 0.5 units below ambient [31] exceed natural swings of pH in other reef areas which show diurnal changes between 7.9 and 8.1 [72]. Weekly to seasonal changes between upwelling and non-upwelling periods in the eastern tropical Pacific are generally below 0.2 pH units [73]. The step changes in pH conditions due to LAIW in our study may thus require an especially high energetic effort to sustain the microenvironment beneath the coral tissue needed for calcification [9], [12]. As *P. lutea* is mixotrophic, LAIW-enhanced fluxes of both inorganic and organic materials may provide additional energy [45], [72], as suggested by the higher nutritional status of *P. lutea* in W compared to E [39]. Similarly, Leichter and Genovese [34] reported positive growth of the coral *Madracis mirabilis* under the influence of daily upwelling due to internal waves and suggested that the lower light conditions might be offset by the higher input of particle and nutrient fluxes. However, the surplus energy in our study does not seem to be sufficient to keep calcification at comparable levels, and the drop in calcification in W 20 m shows that the positive LAIW effects (higher nutritional status of the corals) are outweighed by the negative effects of i.a. temperature and pH. The effect may be much stronger in other corals, particularly the species rare or absent in W [31], as suggested by numerous studies describing massive *Porites* as highly tolerant to different environmental stressors including sedimentation, high nutrition and low pH conditions [21], [74]–[76].

### Bioerosion

Bioerosion was highest in E and lowest in W (Fig. 6) and showed an inverse relationship with temperature anomaly (Fig. 7) refuting our hypothesis of LAIW- (i.e. nutrient-) enhanced bioerosion. However, bioerosion has also been shown to be related to coral cover [2], [14], in line with the observation of higher reef development in E [31], [32]. This is because higher coral cover provides more habitats for internal and external bioeroders, notably living space for boring mussels and sponges, and hiding space for a wealth of external bioeroders such as sea-urchins and fish [2].

The estimation of bioerosion as carbonate loss always implicates possible sources of error due to the difficult estimation of the simultaneously occurring carbonate accretion [5]–[8], [17]–[19]. Internal carbonate accretion on bore holes and channels as well as accretion due to coralline algae was neglected. The comparatively short exposure time of less than 2 years and the marginal actual accretion coming with it justified this approach [5], [7]. The calculation of the skeletal volumes and densities of the newly grown carbonate structures [57] implicated the risk of false estimates because we purely calculated the growth and did not directly measure it. Nevertheless for this study it was the most appropriate and exact method in contrast to direct weight measurements because it was impossible to accurately separate the newly built carbonate structures from the original skeletal blocks. As biological carbonate accretion on the skeletal blocks was shown to be negligible compared to bioerosion (Fig. 6, S6) [5], [7], [19] the possible methodical errors were likely equally insignificant.

Erosion rates in E for both exposure periods and in W shallow after 21 months were similar or higher than rates measured elsewhere in offshore coral reefs denoted as healthy and balanced in their carbonate budget with grazing fish as the main bioeroders [5], [7], [8], [18]. Bioerosion in W 20 m was low even after 21 months exposure contrasting findings in the eastern tropical Pacific where external bioerosion was a function of upwelling intensity [19]. The better light conditions in 7 m depth may explain the higher bioerosion compared to the 20 m sites, where light may have enhanced both, internal bioerosion by favouring phototrophic microborers [77] but also external bioerosion by fostering algal growth [78] and, hence, grazers [77]. After 12 months exposure, skeletal blocks were virtually free of grazing traces and bite marks, and after 21 months W blocks still showed much less external damage than E blocks after 12 months (Fig. S3). Perhaps the duration of the experiment was too short for internal bioerosion to take its toll: in spite of an enhanced supply of plankton [38] likely favouring bioeroding filter feeders [2], [18], [46] it may have taken several months for the boring community to infest the blocks [2], [7], [18]. A lag effect is also suggested by the increase in bioerosion rates after 21 months, only after a community of internal bioeroders may have become established [7].

### Conclusions and outlook

This is the first study indicating evidence for LAIW-depressed coral growth. The results support previous findings that LAIW can inhibit the development of a complex carbonate framework [31], [32] and by this play an important yet largely unexplored role in controlling reef development outside established upwelling regions [19], [23]. They also suggest that surface swell does not suppress coral growth in monsoon areas. Despite the limited growth performance and reduced reef framework coral communities along the W Similans were found to be especially diverse and species rich [31]. This is in contrast to typical upwelling regions [79] or natural CO_2_ vents were only some very robust species dominate the communities [21]. LAIW exposed coral communities therefore offer the possibility to study the tipping points and limits of various coral species to acclimatize or adapt to different natural stress factors simultaneously entrained by LAIW [30], [31], [80] and relevant for future climate scenarios [25]–[27]. Research in this field has to be extended including especially sensitive taxa studying the effects of co-varying stress factors such as temperature, pH and nutrient concentrations. Although the potential of corals to acclimatize still needs to be ascertained, LAIW-exposed corals direct a higher proportion of their energy allocation more to LAIW acclimation rather than growth which may therefore provide an insurance to vagaries of temperature variations in a changing climate.

## Supporting Information

Figure S1 **Microbalance and weighing construction for buoyant weight technique.** (After Davies [36]).
(TIF)

Figure S2 **Coral nubbins of** ***Porites lutea*** **on individual transplant holders.** Nubbins with holders attached to racks built of acrylic glass and metal rocks (a), and coral nubbins on holders right after recollection with epiphytes (b, c) and with epiphytes removed (d).
(TIF)

Figure S3 **Examples for changes on dead coral skeleton during exposure period.** Four different rectangular blocks (ordered among one another) of dead coral skeleton of *Porites lutea* before (East 20 m: A, D, West 20 m: G, J), after 12 months (East 20 m: B, C, West 20 m: E, F) and after 21 months (East 20 m: H, I, West 20 m: K, L) exposure. The second column (B, E, H, K) shows the upper sides, the third column (C, F, I, L) the under sides of the skeletal blocks. The first two rows show blocks right after collection (B, C, E, F), the lower two rows show blocks after drying them (H, I, K, L).
(TIF)

Figure S4 **Bioerosion racks made of PVC-tubes and metal bars.** Each rack with six dead coral blocks of *Porites lutea* attached. View from above the setup (A), and from diagonally below (B).
(TIF)

Figure S5 **Calcium carbonate accretion due to fouling organisms on dead skeletal blocks.** Shown are calcium carbonate precipitating organisms on dead skeletal blocks of *Porites lutea*: from top to bottom: Balanids (A–D), serpulids (E–H), bivalves (I–L), and corals (M–O).
(TIF)

Figure S6 **Comparison of total accretion due to fouling organisms and accretion by different groups of carbonate producers.** Total accretion (upper panels) and accretion by different groups of carbonate producers (lower panels, as fractions of total accretion) on dead coral substrates at island Ko Miang. Error bars: ±1 SE of mean. Results from 12 months exposure (February 2007 to February 2008) (A), and 21 months exposure (February 2007 to November 2008) (B) (number of replicates in each case: 9).
(TIFF)

Table S1 **Analysis of variance (ANOVA) of coral nubbin mortality.**
(DOCX)

Table S2 **Surface area, initial (start) and end air weights of coral nubbins of** ***Porites lutea*** **.**
(DOCX)

Table S3 **Comparison of initial weights of coral nubbins between different sides and depths at Similan Islands.**
(DOCX)

Table S4 **Comparison of measured mass change and calculated mass change of calcium carbonate (CaCO_3_) blocks in east and west of Similan island Ko Miang.**
(DOCX)

Table S5 **Comparison of accretion due to fouling organisms at east (E) and west (W) side of Similan island Ko Miang.**
(DOCX)

Table S6 **Comparison of accretion due to fouling organisms on dead skeletal blocks between 12 and 21 months exposure.**
(DOCX)
