# Supplementary figures and images for: Coral Growth and Bioerosion of Porites lutea in Response to Large Amplitude Internal Waves
Source: PLoS One. 2013 Dec 9;8(12):e73236. doi: 10.1371/journal.pone.0073236 (PMC3867283; doi:10.1371/journal.pone.0073236)

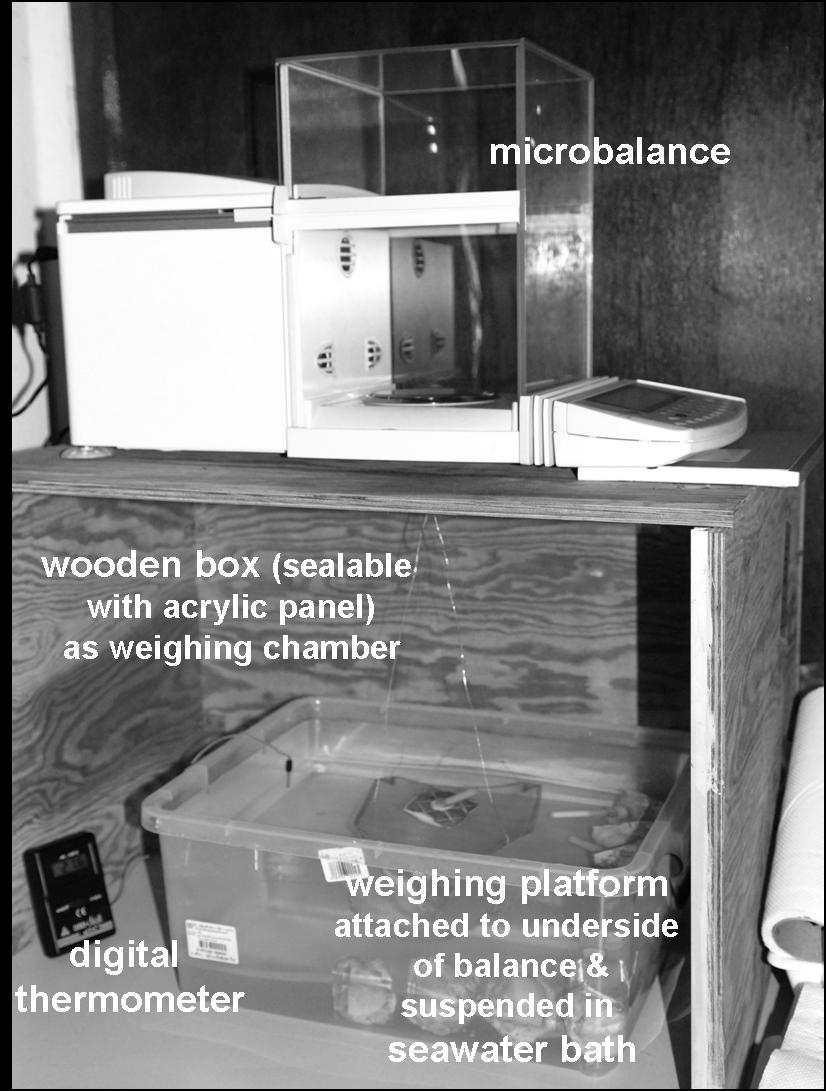

Supplement: Figure S1 — Microbalance and weighing construction for buoyant weight technique. (After Davies [36]). (TIF) [file pone.0073236.s001.tif]

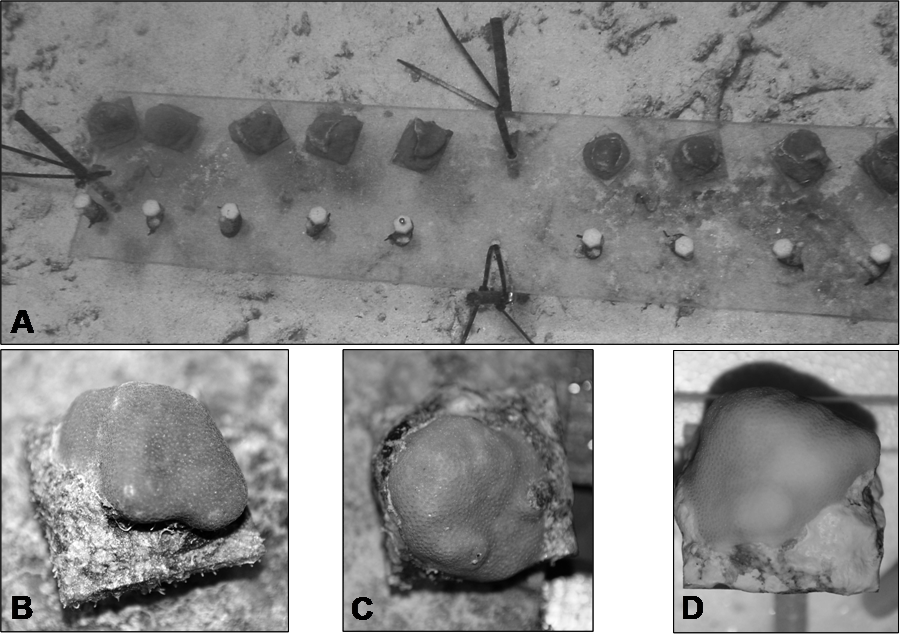

Supplement: Figure S2 — Coral nubbins of Porites lutea on individual transplant holders. Nubbins with holders attached to racks built of acrylic glass and metal rocks (a), and coral nubbins on holders right after recollection with epiphytes (b, c) and with epiphytes removed (d). (TIF) [file pone.0073236.s002.tif]

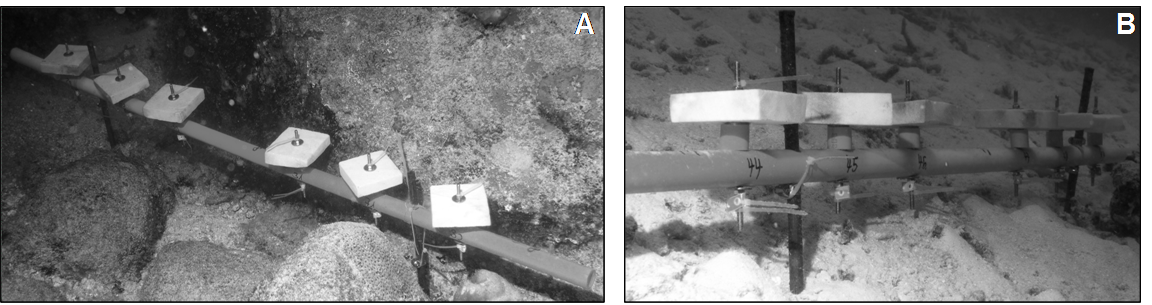

Supplement: Figure S4 — Bioerosion racks made of PVC-tubes and metal bars. Each rack with six dead coral blocks of Porites lutea attached. View from above the setup (A), and from diagonally below (B). (TIF) [file pone.0073236.s004.tif]

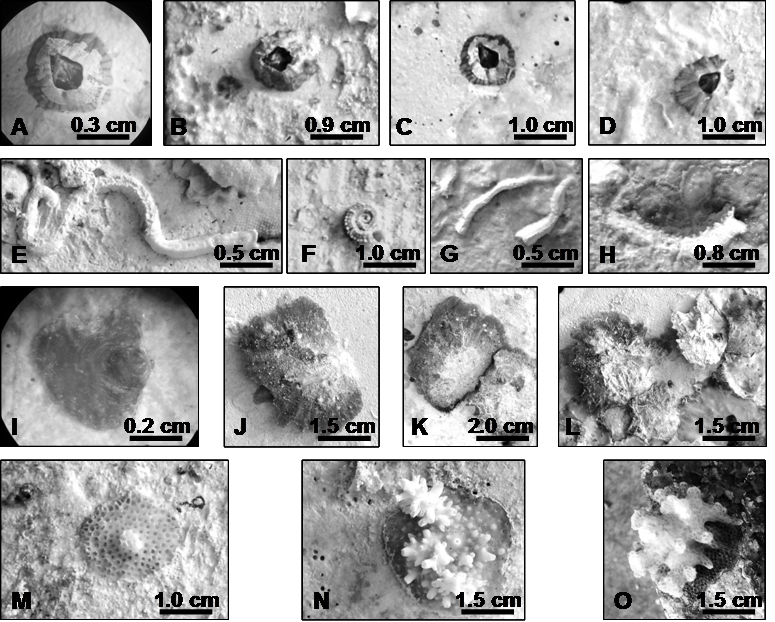

Supplement: Figure S5 — Calcium carbonate accretion due to fouling organisms on dead skeletal blocks. Shown are calcium carbonate precipitating organisms on dead skeletal blocks of Porites lutea: from top to bottom: Balanids (A–D), serpulids (E–H), bivalves (I–L), and corals (M–O). (TIF) [file pone.0073236.s005.tif]

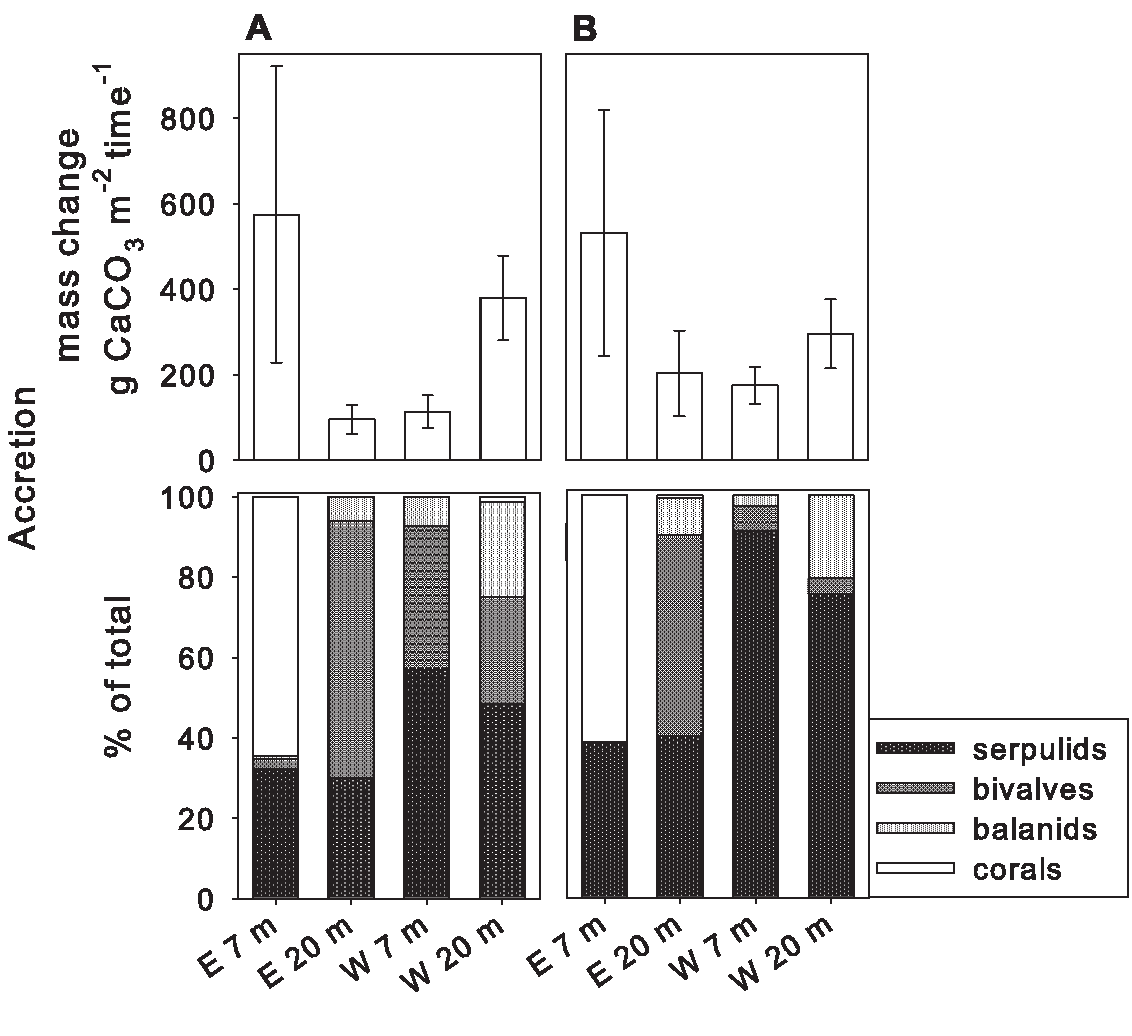

Supplement: Figure S6 — Comparison of total accretion due to fouling organisms and accretion by different groups of carbonate producers. Total accretion (upper panels) and accretion by different groups of carbonate producers (lower panels, as fractions of total accretion) on dead coral substrates at island Ko Miang. Error bars: ±1 SE of mean. Results from 12 months exposure (February 2007 to February 2008) (A), and 21 months exposure (February 2007 to November 2008) (B) (number of replicates in each case: 9). (TIFF) [file pone.0073236.s006.tiff]
