# Supplementary material for: Coral Growth and Bioerosion of Porites lutea in Response to Large Amplitude Internal Waves
Source: PLoS One. 2013 Dec 9;8(12):e73236. doi: 10.1371/journal.pone.0073236 (PMC3867283; doi:10.1371/journal.pone.0073236)
Supplement: Table S1 — Analysis of variance (ANOVA) of coral nubbin mortality. (DOCX) [file pone.0073236.s007.docx]

**Table S1 Analysis of variance (ANOVA) of coral nubbin mortality.**

| Factor | df | MS | F | p |
| --- | --- | --- | --- | --- |
| constant | 1 | 10.31 | 1183.11 | ******* |
| side | 1 | 0.23 | 26.20 | ******* |
| depth | 1 | 0.03 | 3.66 | 0.074 |
| side*depth | 1 | 0.00 | 0.00 | 0.993 |
| Error | 16 | 0.01 |  |  |
| **Tukey HSD**, significantly different, pairwise comparisons: | | | | p |
|  | W 20 m | W 7 m | E 20 m | E 7 m |
| W 20 m |  | n.s. | ***** | n.s. |
| W 7 m | n.s. |  | ******* | ***** |
| E 20 m | ***** | ******* |  |  |
| E 7 m | n.s. | ***** | n.s. |  |

Number of died coral nubbins of *Porites lutea* after transplanting them from a shallow (7 m) sheltered (E) site to 20 Similan Island sites: shallow (7 m, monsoon effect) and deep (20 m, LAIW effect) on the exposed (W) and sheltered (E) sides of 5 different locations in the Similan Islands from February 2007 to February 2008. Side (E and W) and depth (7 and 20 m) as treatment factors; posthoc, pair wise comparisons of the adjusted group means via Tukey HSD-tests. (df = degrees of freedom; MS = means square; F = F-value; p = probability level, significance levels are *0.05 > P ≥ 0.01, ***P < 0.001).
