# Supplementary material for: Coral Growth and Bioerosion of Porites lutea in Response to Large Amplitude Internal Waves
Source: PLoS One. 2013 Dec 9;8(12):e73236. doi: 10.1371/journal.pone.0073236 (PMC3867283; doi:10.1371/journal.pone.0073236)
Supplement: Table S2 — Surface area, initial (start) and end air weights of coral nubbins of Porites lutea . (DOCX) [file pone.0073236.s008.docx]

**Table S2 Surface area, initial (start) and end air weights of coral nubbins of *Porites lutea***.

| **West 20 m** | | | | **West 7 m** | | | |
| --- | --- | --- | --- | --- | --- | --- | --- |
|  | start | | end |  | start | | end |
| island (site) | surface (cm^2^) | weight (g) | weight (g) | island (site) | surface (cm^2^) | weight (g) | weight (g) |
| W4.1 | 9.15 | 14.37 | 18.40 | W7.1 | 16.44 | 19.49 | 33.58 |
| W4.1 | 10.11 | 23.51 | 25.32 | W7.1 | 12.74 | 28.42 | 40.27 |
| W4.1 | 18.99 | 72.71 | 78.89 | W7.1 | 24.08 | 41.29 | 52.58 |
| W4.1 | 7.24 | 26.25 | 36.17 | W7.1 | 22.20 | 30.24 | 53.12 |
| W4.1 | 14.23 | 63.36 | 67.99 | W7.1 | 8.87 | 20.69 | 33.34 |
| W4.1 | 16.93 | 40.97 | 46.29 | W7.1 | 15.73 | 24.44 | 36.24 |
| W4.1 | 13.42 | 41.14 | 45.47 | W7.1 | 33.05 | 38.44 | 60.56 |
| W2.1 | 6.35 | 53.72 | 55.71 | W7.1 | 16.10 | 36.56 | 51.53 |
| W2.1 | 12.14 | 42.54 | 42.89 | W8.2 | 14.10 | 42.06 | 49.98 |
| W2.1 | 14.44 | 44.20 | 49.22 | W8.2 | 7.14 | 15.99 | 19.52 |
| W2.1 | 13.76 | 40.42 | 49.48 | W8.2 | 14.05 | 41.63 | 44.47 |
| W2.1 | 7.62 | 12.96 | 17.89 | W8.2 | 9.87 | 31.87 | 33.76 |
| W2.1 | 6.97 | 28.42 | 29.73 | W8.2 | 10.81 | 25.85 | 26.55 |
| W8.1 | 8.66 | 44.26 | 50.15 |  |  |  |  |
| W8.1 | 5.55 | 31.54 | 41.53 |  |  |  |  |
| W8.1 | 9.97 | 35.49 | 36.60 |  |  |  |  |
| W8.1 | 7.73 | 38.35 | 49.61 |  |  |  |  |
| W8.1 | 9.62 | 39.80 | 45.46 |  |  |  |  |
| W8.1 | 20.91 | 10.57 | 14.81 |  |  |  |  |
| **East 20 m** | | | | **East 7 m** | | | |
|  | start | | end |  | start | | end |
| island (site) | surface (cm^2^) | weight (g) | weight (g) | island (site) | surface (cm^2^) | weight (g) | weight (g) |
| E4.1 | 22.28 | 47.76 | 57.94 | E4.1 | 19.79 | 53.39 | 76.32 |
| E4.1 | 18.28 | 38.23 | 41.43 | E4.1 | 18.57 | 15.84 | 16.55 |
| E4.1 | 9.31 | 26.29 | 28.47 | E4.1 | 25.40 | 51.48 | 77.30 |
| E2.1 | 21.58 | 28.68 | 43.02 | E4.1 | 21.76 | 22.95 | 42.68 |
| E2.1 | 29.98 | 30.53 | 40.52 | E4.1 | 31.14 | 44.85 | 58.83 |
| E2.1 | 29.36 | 31.43 | 49.41 | E4.1 | 45.02 | 51.30 | 88.01 |
| E2.1 | 15.08 | 50.09 | 52.96 | E2.1 | 9.41 | 50.16 | 57.28 |
| E2.1 | 34.48 | 48.42 | 66.14 | E2.1 | 4.12 | 23.27 | 28.29 |
| E2.1 | 23.39 | 11.35 | 35.29 | E2.1 | 17.64 | 39.07 | 46.39 |
| E2.1 | 20.33 | 37.44 | 54.63 | E2.1 | 13.62 | 34.47 | 46.38 |
| E2.1 | 21.17 | 20.61 | 21.79 | E2.1 | 32.03 | 40.50 | 55.06 |
| E7.1 | 12.33 | 40.57 | 73.34 | E2.1 | 25.04 | 32.77 | 54.12 |
| E7.1 | 45.86 | 40.51 | 59.55 | E2.1 | 5.96 | 62.06 | 64.34 |
| E7.1 | 30.24 | 66.14 | 87.26 | E2.1 | 17.79 | 16.67 | 24.98 |
| E7.1 | 39.77 | 52.09 | 63.48 | E7.1 | 4.83 | 26.81 | 27.92 |
| E7.1 | 25.89 | 39.67 | 39.83 | E7.1 | 16.69 | 32.51 | 42.51 |
| E7.1 | 25.39 | 32.28 | 33.88 | E7.1 | 13.78 | 22.09 | 24.37 |
| E7.1 | 24.17 | 16.83 | 24.59 | E7.1 | 22.12 | 31.97 | 44.73 |
| E7.1 | 21.52 | 43.13 | 52.00 | E7.1 | 22.00 | 29.38 | 38.75 |
| E8.1 | 15.75 | 27.72 | 36.59 | E8.1 | 32.72 | 57.13 | 91.63 |
| E8.1 | 17.38 | 45.87 | 49.97 | E8.1 | 24.28 | 56.77 | 91.58 |
| E8.1 | 12.21 | 42.43 | 64.52 | E8.1 | 18.71 | 10.20 | 19.70 |
| E8.1 | 19.62 | 36.63 | 42.58 | E8.1 | 22.14 | 55.59 | 58.65 |
| E8.1 | 11.08 | 28.52 | 33.87 | E8.1 | 25.36 | 33.95 | 67.73 |
| E8.1 | 9.85 | 19.46 | 32.45 | E8.1 | 18.64 | 30.62 | 44.58 |
| E8.1 | 15.81 | 50.92 | 62.83 | E8.2 | 19.08 | 40.02 | 48.81 |
| E8.2 | 25.24 | 50.57 | 84.34 | E8.2 | 14.23 | 69.27 | 76.52 |
| E8.2 | 53.23 | 51.71 | 67.65 |  |  |  |  |
| E8.2 | 16.34 | 44.08 | 56.99 |  |  |  |  |
| E8.2 | 38.81 | 58.12 | 88.05 |  |  |  |  |
| E8.2 | 34.58 | 31.89 | 38.83 |  |  |  |  |
| E8.2 | 30.22 | 57.50 | 87.33 |  |  |  |  |
| E8.2 | 47.47 | 27.98 | 42.39 |  |  |  |  |

Data given for nubbins which were still alive at the end of the experiments. Exposure from February 2007 to February 2008 at different island sites in east (E) and west (W) along Similan Islands (cf. Fig 1).
