# Supplementary material for: Coral Growth and Bioerosion of Porites lutea in Response to Large Amplitude Internal Waves
Source: PLoS One. 2013 Dec 9;8(12):e73236. doi: 10.1371/journal.pone.0073236 (PMC3867283; doi:10.1371/journal.pone.0073236)
Supplement: Table S3 — Comparison of initial weights of coral nubbins between different sides and depths at Similan Islands. (DOCX) [file pone.0073236.s009.docx]

**Table S3 Comparison of initial weights of coral nubbins between different sides and depths at Similan Islands.**

| group 1 versus group 2 | t-value | N group 1 | N group 2 | df | p |
| --- | --- | --- | --- | --- | --- |
| W 20 m versus W 7 m | 1.10 | 19 | 13 | 30 | 0.281 |
| W 20 m versus E 20 m | -0.64 | 19 | 33 | 50 | 0.524 |
| W 20 m versus E 7 m | -0.30 | 19 | 27 | 44 | 0.767 |
| W 7 m versus E 20 m | -2.08 | 13 | 33 | 44 | 0.065 |
| W 7 m versus E 7 m | -1.49 | 13 | 27 | 38 | 0.143 |
| E 20 m versus E 7 m | 0.33 | 33 | 27 | 58 | 0.740 |

Student’s t-test results of nubbins (data square-root-transformed) with all sides (east, E and west, W) and depths (7 and 20 m) included; exposure period from February 2007 to February 2008. (df = degrees of freedom; N = number of samples; t = t-value; p = probability level).
