# Supplementary material for: Coral Growth and Bioerosion of Porites lutea in Response to Large Amplitude Internal Waves
Source: PLoS One. 2013 Dec 9;8(12):e73236. doi: 10.1371/journal.pone.0073236 (PMC3867283; doi:10.1371/journal.pone.0073236)
Supplement: Table S4 — Comparison of measured mass change and calculated mass change of calcium carbonate (CaCO3) blocks in east and west of Similan island Ko Miang. (DOCX) [file pone.0073236.s010.docx]

**Table S4 Comparison of measured mass change and calculated mass change of calcium carbonate (CaCO_3_) blocks in east and west of Similan island Ko Miang.**

| **A** | **12 months exposure** | | | | | |
| --- | --- | --- | --- | --- | --- | --- |
|  | B_gross_ versus B_net_ | t-value | B_gross_ | B_net_ | df | p |
|  | E 7 m | 0.749 | -3.098 | -3.672 | 16 | 0.465 |
|  | E 20 m | 0.254 | -0.296 | -0.391 | 16 | 0.728 |
|  | W 7 m | 0.226 | 0.18 | 0.069 | 16 | 0.824 |
|  | W 20 m | 2.948 | 0.377 | -0.002 | 16 | < 0.01** |
| **B** | **21 months exposure** | | | | | |
|  | B_gross_ versus B_net_ | t-value | B_gross_ | B_net_ | df | p |
|  | E 7 m | 0.552 | -8.333 | -8.969 | 16 | 0.589 |
|  | E 20 m | 0.169 | -3.699 | -3.941 | 16 | 0.868 |
|  | W 7 m | 0.127 | -5.442 | -5.651 | 16 | 0.900 |
|  | W 20 m | 0.435 | -1.401 | -1.633 | 16 | 0.669 |

Measured (B_gross_) and calculated mass change (B_net_, corrected for accretion due to fouling organisms) on dead skeletal blocks exposed in east (E) and west (W) of Similan island Ko Miang in 7 and 20 m depth. Student’s t-test results of skeletal blocks exposed for 12 months (a) and 21 months (b); (df = degrees of freedom; t = t-value; p = probability level, significance level is **0.01 > P ≥ 0.001; N = 9 for all variables compared, mean values given as kg CaCO_3_ m^-2^).
