# Supplementary material for: Coral Growth and Bioerosion of Porites lutea in Response to Large Amplitude Internal Waves
Source: PLoS One. 2013 Dec 9;8(12):e73236. doi: 10.1371/journal.pone.0073236 (PMC3867283; doi:10.1371/journal.pone.0073236)
Supplement: Table S5 — Comparison of accretion due to fouling organisms at east (E) and west (W) side of Similan island Ko Miang. (DOCX) [file pone.0073236.s011.docx]

**Table S5** **Comparison of accretion due to fouling organisms at east (E) and west (W) side of Similan island Ko Miang.**

| **A** | **12 months exposure** | | |  |  |  |  |  |
| --- | --- | --- | --- | --- | --- | --- | --- | --- |
|  |  |  |  | total | accretion by | | | |
|  | significance levels | | | accretion | serpulids | bivalves | balanids | corals |
|  | Kruskal-Wallis test | | | ***** | n.s. | n.s. | ******* | ***** |
|  | W 20 m | vs | E 7 m | n.s. | n.s. | n.s. | ***** | n.s. |
|  | W 20 m | vs | E 20 m | ***** | n.s. | n.s. | ****** | n.s. |
|  | W 20 m | vs | W 7 m | ***** | n.s. | n.s. | ***** | n.s. |
| **B** | **21 months exposure** | | |  |  |  |  |  |
|  |  |  |  | total | accretion by | | | |
|  | significance levels | | | accretion | serpulids | bivalves | balanids | corals |
|  | Kruskal-Wallis test | | | n.s. | n.s. | n.s. | ***** | ***** |

Non-parametric Kruskal-Wallis ANOVA and median test followed by multiple comparisons of mean ranks with side (E and W) and depth (7 and 20 m) as treatment factors. (p = probability level, significance levels are *0.05 > P ≥ 0.01, **0.01 > P ≥ 0.001, ***P < 0.001, n.s.: not significant). Results for experimental period from February 2007 to February 2008, 12 months (a), and from February 2007 to November 2008, 21 months (b).
