# Supplementary material for: Coral Growth and Bioerosion of Porites lutea in Response to Large Amplitude Internal Waves
Source: PLoS One. 2013 Dec 9;8(12):e73236. doi: 10.1371/journal.pone.0073236 (PMC3867283; doi:10.1371/journal.pone.0073236)
Supplement: Table S6 — Comparison of accretion due to fouling organisms on dead skeletal blocks between 12 and 21 months exposure. (DOCX) [file pone.0073236.s012.docx]

**Table S6 Comparison of accretion due to fouling organisms on dead skeletal blocks between 12 and 21 months exposure.**

| **12 months versus** |  |  |  |  |  |
| --- | --- | --- | --- | --- | --- |
| **21 months** | z-value | 12 months | 21 months | U | p |
| **E 7 m** | | | | | |
| total accretion | 0.309 | 0.575 | 0.532 | 44 | 0.757 |
| serpulids | 0.662 | 0.185 | 0.204 | 48 | 0.508 |
| bivalves | 0.574 | 0.014 | 0.000 | 47 | 0.566 |
| balanids | 0.386 | 0.005 | 0.002 | 46 | 0.627 |
| corals | 0.000 | 0.370 | 0.326 | 40 | 1.000 |
| **E 20 m** | | | | | |
| total accretion | 0.839 | 0.095 | 0.204 | 50 | 0.402 |
| serpulids | 0.751 | 0.029 | 0.082 | 49 | 0.453 |
| bivalves | 0.574 | 0.061 | 0.102 | 47 | 0.566 |
| balanids | 0.397 | 0.006 | 0.019 | 45 | 0.691 |
| corals | 0.000 | 0.000 | 0.000 | 40 | 1.000 |
| **W 7 m** | | | | | |
| total accretion | 1.280 | 0.114 | 0.175 | 55 | 0.200 |
| serpulids | 1.987 | 0.065 | 0.160 | 63 | 0.046 |
| bivalves | 0.839 | 0.041 | 0.011 | 50 | 0.402 |
| balanids | 0.397 | 0.008 | 0.004 | 45 | 0.691 |
| corals | 0.691 | 0.000 | 0.000 | 40 | 1.000 |
| **W 20 m** | | | | | |
| total accretion | 1.104 | 0.380 | 0.296 | 53 | 0.270 |
| serpulids | 0.397 | 0.184 | 0.223 | 45 | 0.691 |
| bivalves | 0.662 | 0.101 | 0.012 | 48 | 0.508 |
| balanids | 1.236 | 0.089 | 0.061 | 54 | 0.216 |
| corals | 0.397 | 0.005 | 0.000 | 45 | 0.691 |

Mann-Whitney-U test results of total accretion rates and accretion rates by different groups of carbonate producers after exposure for 12 months (February 2007 to February 2008) and 21 months (February 2007 to November 2008) at Similan island Ko Miang at east (E) and west (W) side in 7 m and 20 m depth. (U = U-value; p = probability level; N = 9 for all variables compared, mean values given as kg CaCO_3_ m^-2^).
